# Leadership Development in Women STEM Students: The Interplay of Task Behaviors, Self-Efficacy, and University Training

**DOI:** 10.3390/bs14111087

**Published:** 2024-11-13

**Authors:** Giuliani Coluccio, Sebastián Muñoz-Herrera, Elisa Adriasola, Elizabeth Escobar

**Affiliations:** 1 Facultad de Ingeniería, Universidad de Tarapacá, Arica 1000000, Chile; 2 Faculty of Engineering, Universidad del Desarrollo, Concepción 4030000, Chile; 3 Facultad de Administración y Economía, Universidad Diego Portales, Huechuraba 8170641, Chile; 4 Business School, The University of Western Australia, Crawley, WA 6009, Australia

**Keywords:** women leadership, higher education, leader prototype, structural equation model

## Abstract

This study explores the relationship between task-oriented behaviors, self-efficacy, and leadership emergence in women STEM students, grounded in the context of prototypical leadership theory and self-efficacy theory. Prototypical leadership theory emphasizes the alignment of leadership behaviors with group expectations, which, in STEM fields, are often task-oriented. The research examines how task-oriented behaviors, such as planning, decision-making, and supervision, influence women’s self-perception of leadership ability and their subsequent emergence as leaders. Our results show a positive relationship between task-oriented behaviors and self-efficacy and a positive relationship between self-efficacy with leader emergence, with academic experience further ngthening this link. As students’ progress through their programs, engaging in more teamwork and leadership tasks, their self-efficacy enhances, leading to stronger leadership emergence. Also, we found an indirect effect from task-oriented behavior to leader emergence via self-efficacy. These findings have significant implications for fostering leadership in women, particularly in STEM. The study calls for educational programs to enhance opportunities for women to develop these behaviors early on, ensuring their growth into leadership roles in STEM fields.

## 1. Introduction

Leadership has been a prominent subject in organizational behavior for a long time, with a strong focus on how leadership behaviors are expressed, cultivated, and perceived in various settings. In the conventional view, leadership has frequently been characterized in terms of gendered expectations, wherein male leaders are usually linked to task-oriented behaviors, while female leaders are more often associated with relational or communal behaviors. However, this dichotomous understanding of leadership behaviors may overlook the complexity and adaptability of women leaders, especially in fields where task-based leadership is crucial, such as science, technology, engineering, and mathematics (STEM). In these contexts, women are increasingly stepping into leadership roles that require them to exhibit strong task-oriented behaviors, challenging conventional leadership prototypes and expanding the understanding of effective leadership in STEM [1].

The prototypical leadership theory suggests that leadership behaviors are typically judged in relation to an implicit standard of an “ideal” leader, which historically leans towards behaviors commonly associated with males, such as decisiveness, assertiveness, and task completion [2,3]. The presence of this prototype is especially pronounced in male-dominated fields like STEM. This might be in part because the emphasis on task-oriented behaviors aligns with the technical and problem-solving aspects of the work. However, as women increasingly pursue careers and leadership roles within STEM, they are compelled to develop and exhibit these task-oriented behaviors to meet the demands of their roles effectively. This phenomenon suggests a shift in the leadership prototype, particularly within the STEM context, where women leaders must navigate and possibly redefine these expectations to succeed [4].

Universities play a crucial role in cultivating leadership skills, particularly for women in STEM fields. Higher education institutions serve as critical environments where leadership behaviors are cultivated, often through both formal education and informal socialization processes [5]. In engineering, science, and technology programs, where the curriculum is heavily task-focused, women students are often required to demonstrate competencies that align with task-oriented leadership from an early stage. This educational context not only prepares women for the technical demands of their future careers but also shapes their leadership styles to be more task-focused, challenging traditional gender norms and leadership prototypes [6]. Furthermore, research has shown that participation in STEM-related university programs and extracurricular activities, such as leadership workshops and project-based learning, can significantly influence the development of leadership skills among women, encouraging a shift towards task-oriented behaviors [7].

Recent studies highlight that the university setting can play a pivotal role in breaking down gendered leadership stereotypes. By providing women in STEM with opportunities to lead project teams, engage in problem-based learning, and participate in leadership development programs, universities help to cultivate a leadership identity that integrates task-oriented behaviors. These experiences can empower women to assert themselves in leadership roles and challenge the conventional belief that task-oriented leadership is inherently masculine [8,9]. The process of cultivating these leadership behaviors within the university context not only prepares women for professional success but also facilitates broader changes in how leadership is understood in traditionally male-dominated domains.

This research paper aims to explore how women in STEM disciplines develop task-based leadership behaviors, particularly within the context of their university education. By examining the experiences of female leaders in engineering, science, and technology, this study seeks to understand how these women navigate the expectations of task-oriented leadership and how their behaviors align with or challenge existing leadership prototypes. Through this exploration, the study will contribute to a deeper understanding of leadership in STEM, offering insights into how women can effectively lead in task-intensive environments and how universities can better support the development of such leadership behaviors [10,11].

## 2. Theoretical Framework

The prototypical leadership theory serves as a fundamental framework for comprehending individuals’ perceptions and recognition of effective leadership. According to this theory, leadership prototypes are mental models or cognitive schemas that people used to evaluate leaders [12]. These prototypes are often culturally ingrained, emphasizing traits such as assertiveness, decisiveness, and confidence—attributes historically associated with male leaders. Prototypical leadership is crucial in leadership theory because it provides insight into how gender biases are perpetuated within organizational settings. It explains why individuals who align with these prototypes are more likely to be recognized and accepted as leaders, while those who do not may struggle to gain leadership recognition [13].

The importance of prototypical leadership within leadership theory cannot be overstated, as it serves as a lens through which leadership behavior is evaluated and interpreted. Leadership prototypes influence the way followers perceive and respond to leaders, often reinforcing existing power structures within organizations [14,15,16]. For instance, in environments where task-oriented behaviors such as problem-solving and decisiveness are highly valued, individuals who display these behaviors are more likely to be seen as effective leaders. This dynamic is particularly pronounced in male-dominated fields like STEM, where leadership prototypes closely align with traditionally masculine traits. These entrenched prototypes pose significant challenges for women aspiring to leadership positions, placing women in an ambivalent situation. On one hand, they are expected to demonstrate leadership behaviors that align with masculine norms; on the other hand, when they do exhibit these traits, they may face backlash, as these behaviors contradict societal expectations of femininity [17,18,19,20]. This role incongruity not only affects women’s ability to ascend to leadership positions but also influences how their leadership is evaluated when they do attain these roles [20]. As a result, women in leadership are often subject to harsher scrutiny and may need to outperform their male counterparts to receive the same level of recognition [2,20,21].

Despite these challenges, the evolving nature of leadership theory has begun to question the rigidity of prototypical leadership models. Zaccaro [22] presents a viewpoint that expands upon the conventional understanding of leadership prototypes, emphasizing the intricate and multifaceted nature of leadership traits. This author asserts that effective leadership cannot be simplified to a singular set of traits or behaviors, as it encompasses a blend of traits that differ based on the circumstances. This perspective is significant because it acknowledges that leadership effectiveness is not solely determined by how well an individual fits a particular prototype but also by how they adapt their traits and behaviors to different situations. Yukl et al. [23] further contribute to this discussion by proposing a hierarchical taxonomy of leadership behavior that integrates a half-century of research on leadership. This taxonomy serves to categorize leadership behaviors into three overarching meta-categories: task-oriented, relations-oriented, and change-oriented behaviors. The central conclusion is that optimal leadership entails a careful integration of these behaviors, considering the specific situational demands. This approach aligns with the idea that leadership is contextually dependent and that rigid adherence to traditional leadership prototypes may limit the effectiveness of leaders in dynamic and complex environments.

The shift towards viewing leadership as more contextual has significant implications for women in leadership roles, particularly in STEM fields. As leadership theory evolves to recognize the importance of adaptability and contextual awareness, there is growing support for a broader range of leadership behaviors that challenge traditional prototypes. For instance, Zaccaro’s [22] emphasis on the complexity of leadership traits suggests women can be effective leaders by leveraging a diverse set of traits that may not fit the traditional masculine prototype but are well-suited to the specific challenges of their roles. Similarly, Yukl et al.’s [23] taxonomy highlights the value of integrating the different behaviors, offering women leaders the flexibility to navigate the unique demands of their environments. This evolving understanding of leadership has also led to the development of new leadership theories that emphasize the importance of context. Contingency theories, for example, argue that the effectiveness of leadership behaviors depends on the specific situational variables such as the nature of the task, the characteristics of the followers, and the organizational culture [24,25]. These theories challenge the notion that there is a one-size-fits-all approach to leadership and instead advocate for a more nuanced understanding of how different leadership behaviors can be effective in different contexts.

Recognition of leadership as a contextual phenomenon aligns with contemporary discussions on diversity and inclusion in leadership. As organizations become more diverse, there is an increasing need to recognize and value different leadership styles that reflect the varied experiences and perspectives of leaders from different backgrounds [19,26,27]. This perspective challenges traditional, often narrow, definitions of leadership that masculine prototypes have historically dominated and instead encourages a more inclusive understanding of leadership that accommodates a broader range of behaviors and traits. This shift is particularly important for women in leadership, as it provides an opportunity to redefine leadership in ways that are more inclusive and representative of the diverse ways in which leadership can manifest. Consequently, the emergence of leadership behaviors in women, especially in male-dominated fields like STEM, becomes a critical area of focus.

The emergence of leadership behaviors is a dynamic process shaped by the interaction of individual traits, contextual influences, and environmental expectations. In STEM fields, where leadership is often tied to technical expertise, problem-solving, and task-oriented behaviors, the path to leadership for women is particularly complex. Women in these areas must navigate the dual challenge of demonstrating technical competence while also meeting social expectations that often align leadership with masculine prototypes [9,17]. As they cultivate leadership behaviors, women in STEM may need to develop task-oriented behaviors—such as decision-making, planning, and supervision—to respond to the environmental expectative in STEM works. Women in STEM who report high levels of these behaviors could demonstrate their ability to achieve tangible outcomes, manage complex tasks, and ensure that their teams operate smoothly and effectively. These task-oriented behaviors not only meet the technical demands of the field but also enhance the leader’s authority, credibility, and influence.

Moreover, the consistent engagement in task-oriented behaviors significantly contributes to the development of self-efficacy. Self-efficacy refers to the belief in one’s ability to perform specific tasks, and it is built through mastery experiences, vicarious learning, and feedback [28,29,30]. The ability of women studying STEM subjects to effectively execute task-oriented behaviors plays a vital role in strengthening their belief in their own abilities and expertise, enabling them to overcome the challenges they encounter during their academic and professional journeys. As they manage and complete projects, provide supervision, and clarify roles within their teams, these experiences of mastery directly enhance their self-efficacy, enabling them to tackle increasingly complex tasks with confidence [31,32,33]. This positive reinforcement of self-efficacy through task-oriented behaviors becomes critical as women progress through their university experience, positioning them to feel more confident in their leadership abilities. In this way, we expect the following relation:

**Hypothesis** **1:** *Higher task-oriented behaviors are positively related to higher levels of self-efficacy in women students in STEM.*

Self-efficacy is a crucial determinant of leader emergence, particularly for women students in STEM fields, where they face unique challenges, including gender biases. Self-efficacy theory emphasizes that individuals who believe in their capability to execute tasks effectively are more likely to exhibit persistence, effort, and resilience—qualities essential for leadership [30]. High self-efficacy among women students in STEM has significant implications, as it empowers them to navigate complex situations successfully and assume leadership roles that demand confidence in their technical skills and decision-making abilities [34,35]. As they engage in academic projects, research, and collaborative tasks, their self-efficacy strengthens through repeated mastery experiences, which are critical to their growth as emergent leaders [32,33,36]. Women students who believe in themselves can defy stereotypes and become strong leaders. This self-belief empowers them to lead teams, tackle complex problems, and show resilience in tough times—all signs of leadership [37,38,39]. This self-perception of competence plays a pivotal role in their leadership trajectory, as women students with higher levels of self-efficacy are more likely to assume leadership roles and be perceived as leaders by others. However, while extreme levels of self-efficacy may not always correlate with positive outcomes in performance, fostering self-efficacy is crucial for women in STEM, given the unique challenges posed by the demanding study plans and subject activities that require both technical and leadership competencies. In this way, higher self-efficacy allows them to meet these challenges effectively and confidently transition into leadership roles.

**Hypothesis** **2:** *Higher levels of auto-perception of self-efficacy are positively related to higher levels of leader emergence in women students in STEM.*

The relationship between self-efficacy and leader emergence in women STEM students is moderated by their academic experiences, which evolve throughout their university education. In the initial stages of STEM programs, students primarily focus on individual-based foundational courses (like calculus, algebra, physics, or chemistry), offering limited opportunities for leadership development. However, as they advance to more specialized, teamwork-intensive courses in the latter half of their programs, women students begin to engage in collaborative tasks and leadership roles. These experiences help build self-efficacy through practical application of leadership behaviors, as they lead teams, manage projects, and solve complex problems in group settings. Teamwork methodologies, which are primarily but not exclusively emphasized in the second half of the curriculum, provide the environment needed for women to exercise leadership and enhance their belief in their ability to lead [40,41]. This aligns with Bandura’s theory of self-efficacy [30], where mastery experiences enhance confidence in leadership abilities, particularly in challenging STEM contexts. With increased exposure to leadership-reinforcing experiences, women students accumulate valuable skills and confidence, making the connection between self-efficacy and leader emergence stronger in later academic stages. This progression allows them to assert their leadership potential, challenging traditional stereotypes that align leadership with masculine traits.

**Hypothesis** **3:** *The relationship between self-efficacy and leader emergence in women students is positively moderated by experience, with the relationship being stronger for students who have undergone more extensive training throughout their university experience.*

The relationship between task-oriented behaviors and leader emergence can be explained by the mediating role of self-efficacy, particularly in the context of women students in STEM fields. Task-oriented behaviors are critical in STEM environments, where technical competence and effective project management are highly valued. Women students who consistently demonstrate these behaviors are likely to develop a sense of self-efficacy, individuals who engage in behaviors that lead to mastery experiences, such as successfully managing tasks and solving problems, reinforce their belief in their own abilities, further increasing their confidence to lead. As self-efficacy strengthens, women students are more likely to emerge as leaders, as they perceive themselves as capable of influencing their team and achieving results. Women who report high levels of task-oriented behaviors not only demonstrate leadership capabilities but also develop a strong belief in their ability to lead, which ultimately enhances their emergence as leaders. Therefore, task-oriented behaviors not only support academic success but also play a key role in strengthening women’s belief in their capacity to lead in STEM environments.

**Hypothesis** **4:** *The relationship between task-oriented behavior and leader emergence is mediated by the self-perception of self-efficacy.*

**Figure 1:**
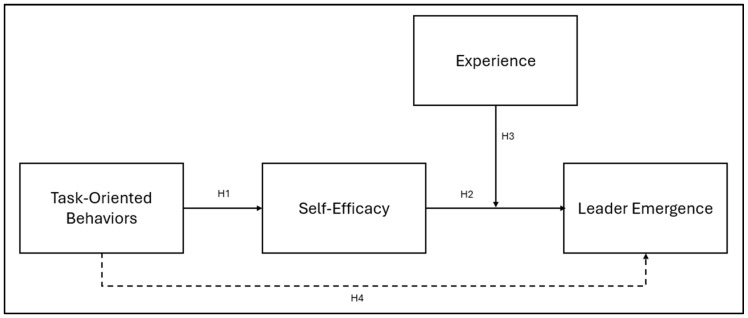
Proposed model.

A resume of the proposed model is shown in Figure 1.

## 3. Materials and Methods

### 3.1. Study Design and Sample

The present study corresponds to a quantitative design that measures the relationships between task-oriented behaviors with the emergence of leadership and the perception of efficacy in women students of industrial engineering. To complete the data collection stage, we apply online questionnaires to students from a Chilean state university. For this study, 114 students answered the questionnaires, representing 49% of the total of women students. Before answering the questionnaire, all the participants provided their informed consent, acknowledging their understanding of the study’s aims according to ethical research standards. After signing to grant their informed consent, the students answered a questionnaire comprising three sections. The first is for control data (identifier, age, and year of admission), the second section captures the auto-perception of task-oriented behaviors and leader emergence, and the third section captures the perception of efficacy.

To address common method bias (CMB), we implemented strategies recommended by Podsakoff et al. [42,43]. Our primary focus was on ensuring respondent anonymity and reducing response apprehension. We guaranteed confidentiality and stressed that all answers were valid. Participants were informed that their data would be aggregated, preventing individual identification. We used anonymous identification codes to protect privacy further. We also explicitly stated that responses would be used solely for research, not individual assessment, to minimize social desirability bias.

These measures were designed to encourage honest responses and reduce potential biases. Furthermore, we utilized validated questionnaires [44,45] known for addressing inherent bias issues. This comprehensive approach aligns with best practices in mitigating CMB and enhancing the validity of self-reported data in organizational research.

### 3.2. Measures

To measure the variables in our study, we employed several established instruments. All instruments used a five-point Likert scale, ranging from 1 (never) to 5 (always). The specific measures are as follows:

▪ Leader emergence: we measured leader emergence through the instrument developed by McCusker [45]. This instrument includes five items based on a self-perception of the frequency with which students believe they develop leadership behavior in different situations. An example item is “I have a high degree of control over the activities of the teams in which I participate”.
▪ Task-Oriented behaviors: we used the instrument developed by McCusker [45] to measure task behaviors. This instrument measures three behaviors: short-term planning, clarification of responsibilities, and supervision of operations and performance, considering three items for each factor. An example of this instrument for each of the dimensions, respectively, is “I organize my team’s tasks in advance and establish deadlines for their completion”, “I make sure that each member of my team is completely clear about what to do and what a way”, and “I care about the progress and quality of the tasks carried out within the team to which I belong”.
▪ Self-efficacy: we used the instrument developed by Doll et al. [44]. This instrument includes three items. An example is “I can rely on my own abilities in difficult situations”.
▪ Experience: the experience of the students was measured by a dichotomous variable. The experience is assigned a value of 1 when the student selects the course in the second half of their career and 0 otherwise.

### 3.3. Plan of Study Description

**Figure 2:**
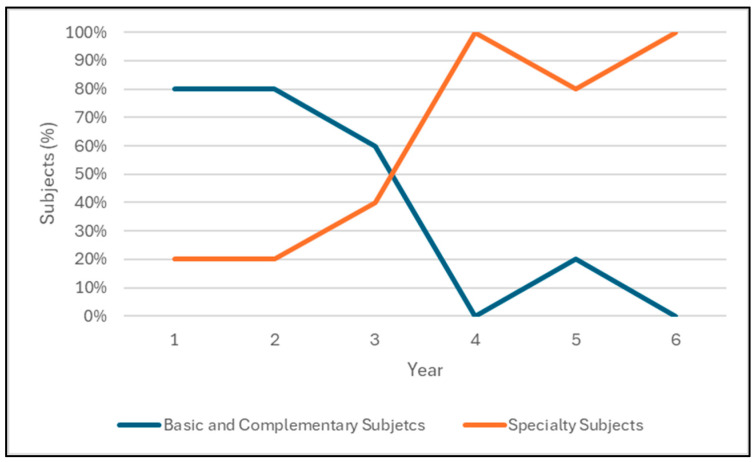
Proportion of subjects.

The industrial engineering degree program that these women are enrolled in comprises a six-year study plan. Hence, the degree encompasses a comprehensive curriculum of 52 subjects, with 28 being dedicated to industrial engineering training and the remaining 24 encompassing a diverse range of subjects in basic sciences and engineering sciences. The study plan emphasizes the development of transversal competences, such as teamwork and oral and written communication, in the specialty subjects. In contrast, the subjects in basic sciences and engineering sciences focus more on building mathematical and physical foundations through autonomous work. Table 1 shows the number of subjects in the degree per year depending on whether it belongs to basic sciences, engineering sciences, general training, or specialty training. Furthermore, as shown in Figure 2, the subjects of the specialty, in proportion, increase over time, going from an average of 27% in the first three years to 93% in the second half of the degree.

## 4. Results

Before examining the proposed relationships, we assessed the internal consistency and reliability of the measured constructs. Table 2 presents Cronbach’s alpha coefficients and descriptive statistics for each construct. The observed Cronbach’s alpha values are deemed appropriate considering the study’s sample characteristics [46]. Descriptive statistics were computed for each variable’s items. These results provide a preliminary indication of the psychometric properties of our measures and offer an overview of the central tendencies and variability in our data.

**Figure 3:**
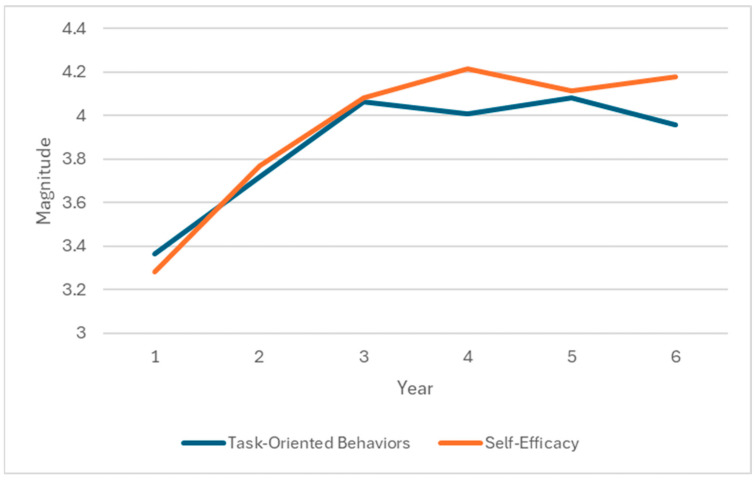
Development of task-oriented behaviors and self-efficacy in women students.

By examining the averages of the students’ studies, one can make a fascinating observation about the development of their task-oriented behaviors and self-efficacy levels. According to Figure 3, task-oriented behaviors and self-efficacy levels exhibit a rising trend resembling an inverted U-shape. Notably, there is significant growth during the initial three years, followed by a more stable progression in the latter half of the course. One explanation for this is that, in the early years, students not only acquire specific competencies but also gradually develop the transversal competencies proposed by the study plan. As a result, they exhibit higher levels of self-efficacy and engage in task-oriented behaviors.

**Figure 4:**
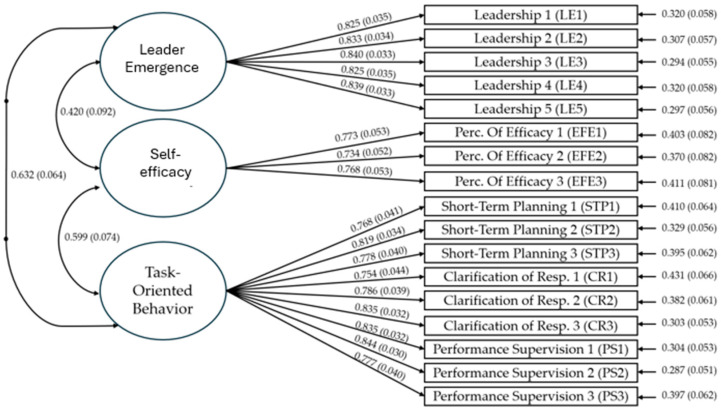
Measurement model.

Subsequently, we conducted a confirmatory factor analysis (CFA) to evaluate the measurement model. In this analysis, all items were specified to load onto their respective latent constructs, and correlations among the latent variables were estimated. The CFA yielded satisfactory fit indices: comparative fit index (CFI) = 0.975, Tucker–Lewis index (TLI) = 0.970, and root mean square error of approximation (RMSEA) = 0.051. These results indicate an adequate fit of the measurement model to the observed data, providing empirical support for the proposed factor structure [47,48,49]. Regarding the convergent and discriminant validity of these constructs, we can point out that task-oriented behaviors (AVE = 0.640; CR = 0.942), leader emergence (AVE = 0.693; CR = 0.919), and perception of efficacy (AVE = 0.606; CR = 0.811) are higher than the cut-off points specified by the literature [47,48,49]. This robust measurement model is a foundation for testing our hypothesized structural relationships. Figure 4 shows the measurement model.

To test our model, first, we calculate the value of task-behavior as the average of the nine items measured. Second, we build the moderator as a dichotomous variable. This variable uses the value of 1 when the age of the student is over 20 and 0 otherwise. We decided on this because we need to include a moderation variable in the SEM as an observed variable to test Hypothesis 2 because the moderation in our model is the multiplication of the task-oriented behavior, and the moderator and the cut-off point were selected because the students are approximately 20 years old and are in the middle of the undergraduate program in the university. Third, for leader emergence and perception of efficacy, we include these variables in the model as a latent variable. Table 3 shows the univariate sample statistics of the observed variables included in the model, and Table 4 shows the estimated correlation matrix of the latent and observed variables included in the model.

The fit index of the model indicates its robustness considering the sample size and variables considered (CFI = 0.92; TLI = 0.91; RMSEA = 0.091). Table 5 presents the results of the SEM, providing insights into the associations between task-oriented behaviors, perception of leader emergence, and perception of efficacy in women STEM students. Our model results show that the relationship between task-oriented behaviors and self-efficacy in women STEM students is positive and significant (β = 0.548; *p* < 0.01), supporting Hypothesis 1. This suggests that women in STEM who engage in more task-oriented behaviors (e.g., planning, problem-solving, and decision-making) tend to develop stronger beliefs in their abilities to succeed in leadership roles. Also, our results show that self-efficacy is positively related to leader emergence (β = 0.342; *p* < 0.01), supporting Hypothesis 2. This indicates that women STEM students who perceive themselves as capable (high self-efficacy) are more likely to emerge as leaders. Our results showed that experience (defined as progressing through more team-oriented and practical components of the STEM curriculum) moderates the relationship between self-efficacy and leader emergence (β = 0.246; *p* < 0.05), supporting Hypothesis 3. In other words, as women students accumulate more experience, the influence of self-efficacy on their ability to emerge as leaders amplifies. Finally, we found that the indirect effect of task-oriented behavior on leader emergence was positive and significant (β = 0.188; *p* < 0.01), supporting Hypothesis 4. Based on this finding, it can be inferred that the development of task-oriented behaviors plays a role in boosting self-efficacy, ultimately leading to more women students in STEM fields becoming leaders.

## 5. Discussion

This study provides insights into factors that support leadership emergence among women students in STEM fields, particularly through the roles of task-oriented behaviors, self-efficacy, and accumulated experience. The findings suggest that task-oriented behaviors foster leadership emergence by enhancing self-efficacy, especially as students progress through their academic programs. Building upon Bandura’s self-efficacy theory [28], This study demonstrates how task-oriented actions contribute to leadership development in a gendered, technical context.

The strong connection between task-oriented behaviors and self-efficacy highlights the importance of active participation in leadership development for women in STEM fields. Female students who engage in task-oriented activities, such as decision-making, planning, and supervision, tend to develop greater self-confidence in their leadership abilities, aligning with the findings of Zaccaro [22] and Yukl et al. [23]. These results not only confirm prior research on the connection between task-oriented behavior and leadership development [50,51,52] but also extended it to the STEM context, where task-oriented behaviors are particularly valued due to their association with technical competence and problem-solving [53,54]. This underscores the need for STEM programs to prioritize these behaviors in order to foster the confidence necessary for women students to emerge as leaders.

In line with this, the study reinforces self-efficacy theory by demonstrating a significant relation between self-efficacy and leader emergence. Students with higher self-efficacy are more likely to perceive themselves as leaders, a finding consistent with prior research on women leaders [55]. This study contributes uniquely by emphasizing the importance of self-efficacy in STEM fields, where leadership often aligns with masculine traits and behaviors [13,17,56,57]. In such environments, women frequently face challenges related to stereotypical expectations, making self-efficacy an essential component in their leadership development. By building confidence through task-oriented behaviors, women students can challenge these norms and position themselves as leaders, further underscoring the importance of self-efficacy in this process.

Additionally, the study shows that experience, operationalized as progression through more advanced and teamwork-oriented courses, moderates the relationship between self-efficacy and leader emergence. As students progress through their academic programs, they transition from individually focused foundational courses to more collaborative, complex projects that emphasize leadership and teamwork. These experiences, encountered primarily in the later stages of their academic journey, strengthen the impact of self-efficacy on leader emergence. This finding supports the notion that women’s leadership development greatly benefits from hands-on leadership experiences, especially in team settings [58,59]. Consequently, it underscores the importance of integrating teamwork methodologies in advanced stages of STEM education to support the growth of leadership abilities.

The findings of this study also reveal the indirect effect of self-efficacy in mediating the relationship between task-oriented behaviors and leader emergence. This aligns with previous research identifying self-efficacy as a key psychological mechanism in leadership development [60,61,62]. Women who engage in task-oriented behaviors gain mastery experiences that reinforce their self-efficacy, thereby increasing the likelihood of leader emergence. This indirect effect highlights the multi-step nature of leadership development for women in STEM, where cultivating task-oriented skills fosters self-efficacy, which, in turn, promotes leadership emergence. This is particularly relevant in STEM fields, where leadership often requires both technical expertise and the confidence to lead teams in high-stakes, performance-driven environments.

Compared with leadership studies, this research offers a more nuanced understanding of leadership emergence by focusing on the specific experiences of women in STEM. While traditional leadership theories often emphasize general traits or behaviors, these findings underscore the importance of contextual factors—such as the nature of STEM education and gendered expectations—that shape leadership development and recognition [22,50,52]. The study also adds to the literature on gender and leadership by providing empirical evidence of how self-efficacy and task-oriented behaviors interact to facilitate leader emergence in a field in which women have been historically underrepresented.

The implications of these findings are significant for leadership theory and practice, particularly in educational settings. Leadership development programs for women in STEM should place a stronger emphasis on cultivating task-oriented behaviors and building self-efficacy through experiential learning. By fostering these components early and consistently throughout students’ academic journeys, institutions can better prepare women for leadership roles, helping to break down traditional gender barriers in STEM fields. Additionally, these findings support the view that leadership is a dynamic, context-specific process shaped by both personal development and environmental influences, aligning with recent calls for more contextualized theories of leadership [22,38,52,63].

The findings of this study suggest that the development of self-efficacy among women in STEM programs is shaped by the unique demands and challenges inherent to these fields. Unlike non-STEM programs, STEM disciplines require technical mastery, task-oriented behaviors, and problem-solving under pressure, which are integral to leadership development in this environment [64]. In non-STEM fields, such as the arts or social sciences, self-efficacy may develop through different pathways, focusing more on interpersonal dynamics, creativity, and adaptability. Although growth in self-efficacy is a common outcome across rigorous academic settings, the distinct features of STEM—including the technical demands and male-dominated culture—make the connection between self-efficacy and leadership especially crucial for women. This unique combination of factors in STEM fields heightens the relevance of targeted support to foster women’s leadership trajectories within these disciplines.

Despite these strengths, this study has certain limitations that must be considered. First, the study relies on self-reported measures of task-oriented behaviors, self-efficacy, and leader emergence, which may introduce response biases such as social desirability bias or inaccuracies in self-assessment [43]. Participants may overestimate or underestimate their leadership abilities or task behaviors, leading to potential distortions in the data. Additionally, due to the study’s cross-sectional design, we are not able to draw causal inferences between task-oriented behaviors, self-efficacy, and leadership emergence, as it only captures a single point in time [65]. Longitudinal studies could offer more comprehensive insights into how these relationships evolve, particularly as women progress through their academic STEM programs and transition into professional roles [66,67].

Nonetheless, a notable strength of this research is the inclusion of women at different stages of their undergraduate program, allowing accumulated experience to serve as a proxy for examining the effect of the proposed relationships over time. Another limitation is the focus on academic experience as a moderating variable, without accounting for broader contextual factors—such as institutional culture, specific STEM program, or societal expectation—and cultural norms which could influence leadership development in women. These external factors can shape self-efficacy and leadership perceptions in ways not fully captured by the study’s design, highlighting the need for future research to explore the interplay between personal, academic, and social influences on leadership emergence in women. We elaborate on some of these issues next.

This study underscores the importance of developing task-oriented behaviors to facilitate leadership emergence in women students in STEM fields. However, it is also essential to acknowledge the broader societal and organizational challenges that women may face when exhibiting these behaviors. Research has shown that women leaders who demonstrate task-oriented or agentic behaviors often encounter negative evaluations, as these behaviors may conflict with traditional gender stereotypes that favor communal attributes in women [17,20,68]. This presents a significant challenge: preparing women to meet the technical and leadership demands of STEM careers while also equipping them to navigate the potential bias and resistance. Future research should explore strategies to balance the development of task-oriented behaviors with addressing the stereotypes that frequently confront women in leadership roles. Understanding how to support women in overcoming these social barriers will be crucial in fostering more inclusive leadership environments. 

Conducting comparative studies with male cohorts alongside female participants would also deepen our understanding of leadership dynamics in STEM settings. A comparative approach could shed light on the distinct challenges women face in leadership roles and evaluate the effectiveness of interventions aimed at enhancing leadership competencies. Longitudinal studies tracking leadership development over the course of academic programs could reveal critical differences in leadership emergence and self-efficacy between genders. Insights gained from such research would not only enrich academic discussions on gender and leadership but also inform the design of targeted programs that promote women’s advancement in STEM fields. These efforts would contribute to creating an inclusive environment that values and supports diverse leadership styles. 

Future research should broaden the examination of leadership emergence among women in STEM by incorporating a wider range of leadership theories, such as transformational, authentic, and servant leadership, alongside traditional theories like transactional leadership. Transformational leadership, which emphasizes inspiration, motivation, and building an emotional connection with followers, aligns closely with traditionally feminine attributes such as collaboration and empathy [9,69]. Authentic leadership, as highlighted by Braun et al. [9], also resonates with communal and relational traits, suggesting that women in STEM might be perceived as more authentic leaders when they exhibit these qualities. In contrast, transactional leadership focuses on structured exchanges between leaders and followers [70] and is often associated with more task-oriented, traditionally masculine characteristics. 

By examining how these different leadership styles manifest in STEM contexts, researchers can gain a better understanding of the unique challenges and opportunities for women in these fields. This approach could contribute to a more nuanced understanding of their leadership trajectories and inform the development of leadership programs that support diverse leadership styles, ultimately creating a more inclusive model of leadership suited to the distinct demands of STEM. 

Finally, this study focused on women’s leadership development in a specific STEM context; it is essential to recognize the influence of cultural dimensions on leadership emergence. Cultural norms related to gender roles suggest that gender egalitarianism and power distance could shape how leadership behaviors are perceived and enacted [71,72]. In the Chilean context, where this study was conducted, cultural norms around gender and leadership likely played a role in shaping leadership perceptions and behaviors. Chile’s cultural landscape can be characterized by strong collectivist values, high power distance, and a marked aversion to uncertainty. Within this framework, moderate emphasis is placed on both gender equality and performance, while humane relationships and quality of life are held in high regard. These cultural traits underscore the importance of social harmony, loyalty, and a deep respect for tradition, alongside the need for structured environments and careful decision-making processes. For women in STEM, this context presents unique challenges, as they must navigate an environment that, while slowly advancing towards gender equality, remains steeped in traditional values. These women face expectations to uphold relational orientations and meet high performance standards within a hierarchical society, where power distance can serve as an additional barrier. 

Preliminary findings from this research suggest that supporting women in STEM as they balance these sometimes-conflicting demands may be critical to fostering their growth as leaders. By building confidence in their leadership abilities and enhancing their technical expertise, women may be better equipped to overcome initial stereotypes that often hinder leadership emergence in STEM fields. This work contributes insights that may empower women in Chile’s STEM industries to navigate and succeed within a traditionally structured and relationship-oriented context. Future research should explore these cultural factors more explicitly by integrating cross-cultural comparisons or measuring cultural orientations to better understand the impact of societal expectations on women’s leadership development. This approach would not only enhance the applicability of the findings across diverse contexts but also offer a more comprehensive view of how culture intersects with gender and leadership in STEM, where stereotypes and challenges extend across borders and cultures. 

## 6. Conclusions

In conclusion, the findings of this study underscore the pivotal role of self-efficacy in the emergence of leadership among women in STEM fields. The findings reveal that task-oriented behaviors significantly enhance women’s self-perception of leadership capabilities, which, in turn, positively influences their emergence as leaders. This relationship is further reinforced as students advance through their academic programs, where exposure to more complex training and teamwork methodologies offers additional opportunities to strengthen self-efficacy and leadership skills. These results align with self-efficacy theory, suggesting that as women in STEM gain experience and confidence, they increasingly perceive themselves as capable leaders, thus bridging the gap between task-oriented behaviors and leadership emergence. 

Moreover, the study highlights the importance of the educational environment in shaping leadership development for women students in STEM. Universities play a key role in creating opportunities for women to engage in task-oriented activities that boost self-efficacy. By fostering environments that support leadership development through practical experiences, mentorship, and collaborative projects, institutions can help women in STEM overcome traditional leadership barriers and embrace leadership roles. The implications of these findings extend to educational policies and leadership training, underscoring the importance of designing interventions that not only enhance technical proficiency but also empower women to recognize and cultivate their leadership potential throughout their academic journey. 

This research provides valuable insights that challenge gender stereotypes, particularly the belief that women are less likely to exhibit task-oriented behaviors. Based on our findings, it is evident that leadership effectiveness can be significantly shaped by external factors rather than depending solely on inherent traits like gender. As Zaccaro [22] points out, aligning behaviors with situational demands is critical for fostering leadership emergence, with certain traits merely predisposing individuals toward leadership roles. By focusing on environmental factors, this study challenges the enduring stereotype that task-oriented leadership is inherently masculine. In STEM fields, leadership does not revolve around gender-specific attributes; rather, it is defined by the skills and behaviors needed to meet situational demands. 

We believe that this situational perspective is particularly relevant in STEM contexts, where success often hinges on technical proficiency and collaborative problem-solving. Here, leadership emerges from an individual’s capability to address specific challenges, rather than their conformity to traditional gender norms. As research has shown, leadership effectiveness is closely linked to individuals’ responsiveness to group or task demands rather than inherent characteristics [50]. By emphasizing the role of environmental factors, our study shifts the narrative away from gendered assumptions about leadership, promoting a more inclusive and capability-driven understanding of leadership potential. 

## Figures and Tables

**Table 1:** Subjects in plan of study.

Year	Basic Sciences	Engineering Sciences	General Formation	Specialty
1	6	1	1	2
2	5	3	0	2
3	2	3	1	4
4	0	0	0	10
5	0	0	2	8
6	0	0	0	2

**Table 2:** Internal reliability and descriptive statistics.

Variable	Mean	SD	Cronbach Alpha
Leader emergence	3.80	0.70	0.92
Task-oriented behaviors	3.82	0.66	0.94
Perception of efficacy	3.88	0.61	0.82

**Table 3:** Univariate sample statistics.

Variable	Mean	Variance	Skewness	Kurtosis	% Min	% Max	Median
LE1	3.851	0.723	−0.310	−0.570	6.14%	23.68%	4.000
LE2	3.781	0.540	−0.295	−0.069	4.39%	14.04%	4.000
LE3	3.711	0.574	−0.069	−0.409	4.39%	14.04%	4.000
LE4	3.772	0.615	−0.231	−0.348	5.26%	16.67%	4.000
LE5	3.877	0.809	−0.335	−0.751	7.02%	28.07%	4.000
STP1	3.772	0.527	−0.035	−0.424	2.63%	14.91%	4.000
STP2	3.868	0.623	−0.084	−0.760	2.63%	22.81%	4.000
STP3	3.912	0.571	−0.219	−0.441	2.63%	21.93%	4.000
CR1	3.816	0.606	−0.336	−0.198	5.26%	17.54%	4.000
CR2	3.930	0.679	−0.245	−0.732	3.51%	27.19%	4.000
CR3	3.886	0.627	−0.112	−0.760	2.63%	23.68%	4.000
OPM1	3.816	0.589	0.093	−0.856	1.75%	20.18%	4.000
OPM2	3.965	0.630	−0.043	−1165	0.88%	28.95%	4.000
OPM3	3.754	0.659	−0.114	−0.585	5.26%	18.42%	4.000
SEFE	3.930	0.389	−0.360	−0.001	0.88%	7.89%	4.000
Self-Efficacy × Experience	2.520	4.142	−0.360	−1733	38.60%	5.26%	3.667

**Table 4:** Estimated correlation matrix.

	Leader Emergence	Task-Oriented Behaviors	Self-Efficacy	Self-Efficacy × Experience
Leader emergence	1.000			
Task-oriented behaviors	0.188 **	1.000		
Self-efficacy	0.342 **	0.548 **	1.000	
Self-efficacy × experience	0.246 *	0.000	0.000	1.000

** *p* < 0.01; * *p* < 0.05.

**Table 5:** Results of the proposed relationships.

Variable	Estimate	S.E.	Est./S.E.	*p*-Value
**Self-Efficacy on**				
Task-oriented behaviors	0.548	0.069	7.991	0.000
**Leader Emergence on**				
Self-efficacy	0.342	0.098	3.482	0.000
Self-efficacy × experience	0.246	0.102	2.415	0.016
**Specific Indirect Effect from Task-Oriented Behavior to Leader Emergence**	0.188	0.060	3.108	0.002

## Data Availability

The original contributions presented in the study are included in the article; further inquiries can be directed to the corresponding author.
